# Study on the intervention effect of mindfulness-based stress reduction on postoperative cognitive dysfunction and psychological resilience in lung cancer patients

**DOI:** 10.3389/fneur.2025.1689318

**Published:** 2025-12-09

**Authors:** Xiaona Ji, Haiyan Ding, Yingtao Meng

**Affiliations:** 1 Ward One of Pulmonary Surgery, Cancer Hospital Affiliated to Shandong First Medical University, Jinan, China; 2 Intensive-Care Unit, Jinan Children's Hospital, Jinan, China; 3 Department of Nursing Management, Cancer Hospital Affiliated to Shandong First Medical University, Jinan, China

**Keywords:** mindfulness-based stress reduction, lung cancer, postoperative cognitive dysfunction, psychological resilience, intervention effect

## Abstract

**Background:**

Postoperative cognitive dysfunction and psychological problems seriously affect the quality of life and recovery of lung cancer patients. As an emerging psychological intervention method, mindfulness-based stress reduction (MBSR) has been widely recognized and applied in the medical field.

**Objectives:**

Evaluating the effectiveness of MBSR as an intervention for postoperative cognitive dysfunction and psychological resilience in lung cancer patients.

**Methods:**

A total of 86 patients who underwent lung cancer surgery in our hospital from January 2022 to December 2023 were enrolled in this study. The research subjects were divided into the control group and the research group by using the method of random number table, each with 43 cases. The control group used conventional care, while the research group used mindfulness-based stress reduction. Differences in outcome indicators between the two groups were assessed through the relevant assessment tools, with statistically significant differences defined as a *p*-value of < 0.05.

**Results:**

Before the intervention, there were no significant differences in cognitive functioning, psychological resilience, self-efficacy, cancer-induced fatigue, and sleep quality scores between the two groups (*p* > 0.05). After 8 weeks of intervention, Montreal Cognitive Assessment (MoCA), Connor-Davidson Resilience Scale (CD-RISC), and General Self-Efficacy Scale (GSES) scores were elevated in both groups, and in the research group, the MoCA scores (26.23 ± 1.45 vs. 25.05 ± 1.17, *p* < 0.001), the CD-RISC total score (66.26 ± 8.27 vs. 61.79 ± 7.93, *p* = 0.012), and GSES score (30.19 ± 3.27 vs. 26.37 ± 2.31, *p* < 0.001) were significantly higher than those of the control group. In addition, Piper Fatigue Scale score (PFS) and Pittsburgh Sleep Quality Index (PSQI) scores decreased in both groups after the intervention. Behavioral fatigue (4.02 ± 1.28 vs. 4.61 ± 1.37, *p* = 0.045), emotional fatigue (3.28 ± 1.39 vs. 3.93 ± 1.40, *p* = 0.033), somatic fatigue (3.81 ± 1.30 vs. 4.47 ± 1.37, *p* = 0.026), and cognitive fatigue (4.07 ± 1.39 vs. 4.72 ± 1.37, *p* = 0.031) were significantly lower in the research group than in those in the control group, as was the total sleep quality score (8.63 ± 1.59 vs. 11.12 ± 1.31, *p* < 0.001).

**Conclusion:**

MBSR can effectively improve postoperative cognitive function, enhance psychological resilience, and alleviate cancer-induced fatigue, and sleep disorders in lung cancer patients.

## Introduction

Lung cancer is one of the malignant tumors with the highest morbidity and mortality rates worldwide (1–3). According to Global Cancer Statistics 2022, there are approximately 2.48 million new cases of lung cancer, accounting for 12.4% of all cancer cases, and approximately 1.8 million deaths, accounting for 18% of all cancer-related deaths (4). With advancement in medical technology, the treatment of lung cancer has become increasingly abundant, including surgery, radiotherapy, chemotherapy, and targeted therapy (5–8). Surgical resection is still the main treatment for lung cancer patients, but postoperative complications and psychological problems have gradually become important factors affecting patients’ prognosis. Among them, postoperative cognitive dysfunction (POCD) is a common postoperative neurological complication in lung cancer patients, which manifests itself as memory loss, decreased attention, and impaired executive function, which seriously affects the patients’ quality of life and recovery process (9–11). Moreover, the trauma of surgery, uncertainty, and side effects of treatment often lead to anxiety, depression, and a decrease in psychological resilience, further exacerbating the physical and psychological burdens (12–15). Psychological resilience refers to an individual’s ability to cope positively and recover when facing adversity, trauma, or significant stress (16). For lung cancer patients, it is crucial to maintain good psychological resilience, which not only helps patients to better cope with the disease and the various difficulties in the treatment process but also promotes their overall physical and psychological recovery.

While traditional nursing care provides essential medical support, it often fails to address the multifaceted psychological and cognitive challenges faced by lung cancer patient’s post-surgery. Currently, there is a lack of structured, evidence-based psychological interventions tailored to this population to alleviate POCD and enhance psychological resilience. MBSR is a new psychological intervention method that has been widely researched and applied in the medical field in recent years (17, 18). By guiding patients to pay attention to the current internal experience and external environment, MBSR cultivates patients’ awareness and acceptance of their own emotions, thoughts, and physical sensations, and helps them to regulate emotions, reduce pressure, and enhance psychological well-being (19). It has been proven that MBSR is effective in the treatment of a variety of chronic diseases and psychological disorders (20–22). It provides a novel approach or a method for improving POCD and enhancing psychological resilience in patients with lung cancer. The purpose of this study was to explore the intervention of MBSR on POCD and the psychological recovery ability of lung cancer patients through a randomized controlled design and related assessment tools. This study aims to deeply analyze the application value and mechanism of positive pressure and decompression therapy in lung cancer patients, thereby providing a theoretical basis and practical guidance for optimizing rehabilitation treatment programs.

## Materials and methods

### General information

A total of 86 lung cancer patients who underwent surgical treatment in our hospital from January 2022 to December 2023 were enrolled in this study. Using the random number table, the subjects were divided into the research group (43 cases) and the control group (43 cases). The control group underwent conventional care, while the research group applied MBSR in addition to conventional care. All patients and their families were fully informed of the study protocol, voluntarily agreed to participate, and signed the informed consent form. The study was reviewed and approved by the Medical Ethics Committee of our hospital.

### Inclusion and exclusion criteria

Inclusion criteria included: (1) confirmed diagnosis in accordance with the relevant lung cancer criteria of the Chinese Medical Association Lung Cancer Clinical Diagnosis and Treatment Guidelines (2018 edition); (2) Karnofsky performance status score ≥70; (3) expected survival time >6 months; (4) patients with good conscious state and communication ability, and reading and writing ability; and (5) complete clinical data. Exclusion criteria included: (1) combination of other malignant tumors or major diseases; (2) physical dysfunction; (3) hearing, vision, speech disorders, and cognitive dysfunction; (4) surgical intolerance; (5) distant metastases; (6) pregnancy or lactation; and (7) previous psychological or psychiatric disorders.

### Intervention

The control group includes patients who receive conventional care. Follow the doctor’s instructions to implement perioperative diagnostic and therapeutic activities; closely observe the patient’s condition changes and vital signs; explain to the patients about lung cancer and surgery; understand the patient’s psychological state through communication, and use verbal communication to patiently guide their emotions; and conduct follow-up visits by telephone every 2 weeks after the patient’s discharge from the hospital to learn about their postoperative recovery and provide targeted health guidance. The intervention period was 8 weeks.

The research group includes patients who apply MBSR intervention along with conventional care. In order to ensure the intervention effect, the research group set the number of participants in each group to be 8–10 cases, which was divided into 5 groups in total, and the patients’ families were encouraged to participate. Lectures were conducted in the activity room of lung cancer surgery in individual groups, with a single course of 2.5 h of explanation and demonstration, 60 min of practice, 30 min of experience sharing, and on-site Q&A and weekly lectures were conducted for a total of 8 weeks.

(1) Mindfulness of breathing (1st and 2nd week): First, introduce the theoretical knowledge related to MBSR to the group members, and talk about the intervention program, process, and main content. Patients should be instructed to take a comfortable position, adjust their breathing, pay attention to how air enters and exits the body, and focus on breathing exercises. Afterward, focus on the body scanning from feet to head, perceive the sensation of different parts of the body, take the body as a whole organism, and feel all kinds of physiological sensations, emotions, and thoughts. During the sensation process, regional tension may occur; there is no need to focus on it; go with the flow and perceive the tension and its diffusion area.
(2) Sitting meditation (3rd and 4th week): The patient sits in a sitting position and starts by feeling the state of breathing, shifting the attention to ideas and thoughts without judgment, and experiencing the state of their occurrence and disappearance. Attention is shifted to the perception of the environment, experiencing movements and sounds in the surroundings, and carefully experiencing the changes in the heart and the body.
(3) Walking Meditation (5th and 6th week): Look carefully at your surroundings and take a few minutes to feel everything around. Then start walking. During the walking process, shift your attention to walking, feel the sensation of the toes, feet, and heels in contact with the ground, the source of force, pay attention to how the arms are swinging and how the legs are walking, walk slowly, and perceive the completion of the process of each walking movement.
(4) Self-exploration (7th and 8th week): First, breathing awareness, then body scanning, during which the patient is gradually exposed to fear, anxiety, or other unpleasant emotions and is allowed to perceive and identify with these emotions and to perceive the body’s feelings about them without having to deal with them. During the process, the patient may discover on his or her own the cause of the unpleasant emotion, begin to identify what was previously unidentified, and begin to give himself or herself enough space for mental activity. Common emotional adjustment strategies are taught to help the patient positively reappraise, accept, and rationally analyze, refocus, and replant.

### Observation indicators

(1) Cognitive function: The Montreal Cognitive Assessment (MoCA) (23) was used to assess patients’ cognitive function. The scale includes eight cognitive domains, including attention and concentration, executive function, memory, language, visual structural skills, abstract thinking, calculation, and orientation, with a total score of 30 and ≥26 as normal. (2) Psychological resilience: The Connor–Davidson Resilience Scale (CD-RISC) (24) was used to assess the psychological resilience of the patients, which consists of three dimensions, namely, resilience, strength, and optimism, with a total score of 100, with higher scores indicating better psychological resilience. (3) Self-efficacy: The general self-efficacy scale (GSES) (25) was used to assess self-efficacy, with a score range of 10–40, which was positively correlated with self-efficacy. (4) Cancer-induced fatigue: The Piper Fatigue Score (PFS) (26) was used to evaluate the cancer-induced fatigue of patients. The scale consists of four dimensions: behavioral, emotional, somatic, and cognitive, and each dimension and the total score of PFS are 10 points, with higher scores indicating more severe fatigue. (5) Sleep quality: The Pittsburgh Sleep Quality Index (PSQI) (27) was used to evaluate the total sleep quality of patients. The scale contains seven components; each component is scored on a 0–3 scale, and the cumulative score of each component is the total PSQI score, which ranges from 0 to 21, and the higher the score, the worse the patient’s sleep quality.

### Statistical analysis

Statistical analysis and plots were performed using GraphPad Prism 8.0. Distributional data were expressed as mean ± standard deviation, and a *t*-test was used for comparison between two groups. Count data were expressed as the number of cases (%), and comparisons between the two groups were performed using the chi-square test, and when the theoretical frequency of the chi-square test was less than 5, the Fisher’s exact test was used. A *p*-value of < 0.05 was taken as the difference was statistically significant.

## Results

### General clinical data

General clinical data of age, gender, body mass index, education level, time of diagnosis, medical history, surgical history, lesion site, and tumor node metastasis (TNM) stage in the research group and the control group were not significantly different by *t*-test, chi-square test, or Fisher’s exact test (*p* > 0.05; Table 1).

**Table 1:** Comparison of the general data for the two groups of patients.

Characteristic	Research group (*n* = 43)	Control group (*n* = 43)	*p*-value
Age (years)	63.44 ± 12.58	63.97 ± 11.71	0.846
Men, *n* (%)	28 (65.12)	25 (58.14)	0.506
BMI, kg/m^2^	23.16 ± 2.33	22.86 ± 2.62	0.573
Educational level, *n* (%)	–	–	0.655
Primary school	20 (46.51)	18 (41.86)	–
Junior high school	10 (23.26)	13 (30.23)	–
Secondary and high school	8 (18.60)	9 (20.93)	–
College, Bachelor’s Degree and above	5 (11.63)	4 (9.30)	–
Time of diagnosis, month, *n* (%)	–	–	0.907
<1	5 (11.63)	7 (16.28)	–
1–3	11 (25.58)	12 (27.91)	–
3–6	10 (23.26)	9 (20.93)	–
>6	17 (39.53)	15 (34.88)	–
Medical history, *n* (%)	–	–	–
Hypertension	15 (34.88)	17 (39.53)	0.656
Diabetes	8 (18.60)	10 (23.26)	0.596
Coronary heart disease	5 (11.63)	4 (9.30)	>0.999
Hypercholesterolemia	2 (4.65)	3 (6.98)	>0.999
Surgical history, *n* (%)	–	–	0.387
No	18 (41.86)	22 (51.16)	–
Yes	25 (58.14)	21 (48.84)	–
Lesion site, *n* (%)	–	–	0.799
Right middle lobe	12 (27.91)	11 (25.58)	–
Right lower lobe	20 (46.51)	17 (39.53)	–
Upper left lobe	8 (18.60)	10 (23.26)	–
Lower left lobe	3 (6.98)	5 (11.63)	–
TNM staging, *n* (%)	–	–	0.532
I	25 (58.14)	21 (48.84)	–
II	15 (34.88)	20 (46.51)	–
III	3 (6.98)	2 (4.65)	–

### Cognitive function

**Figure 1:**
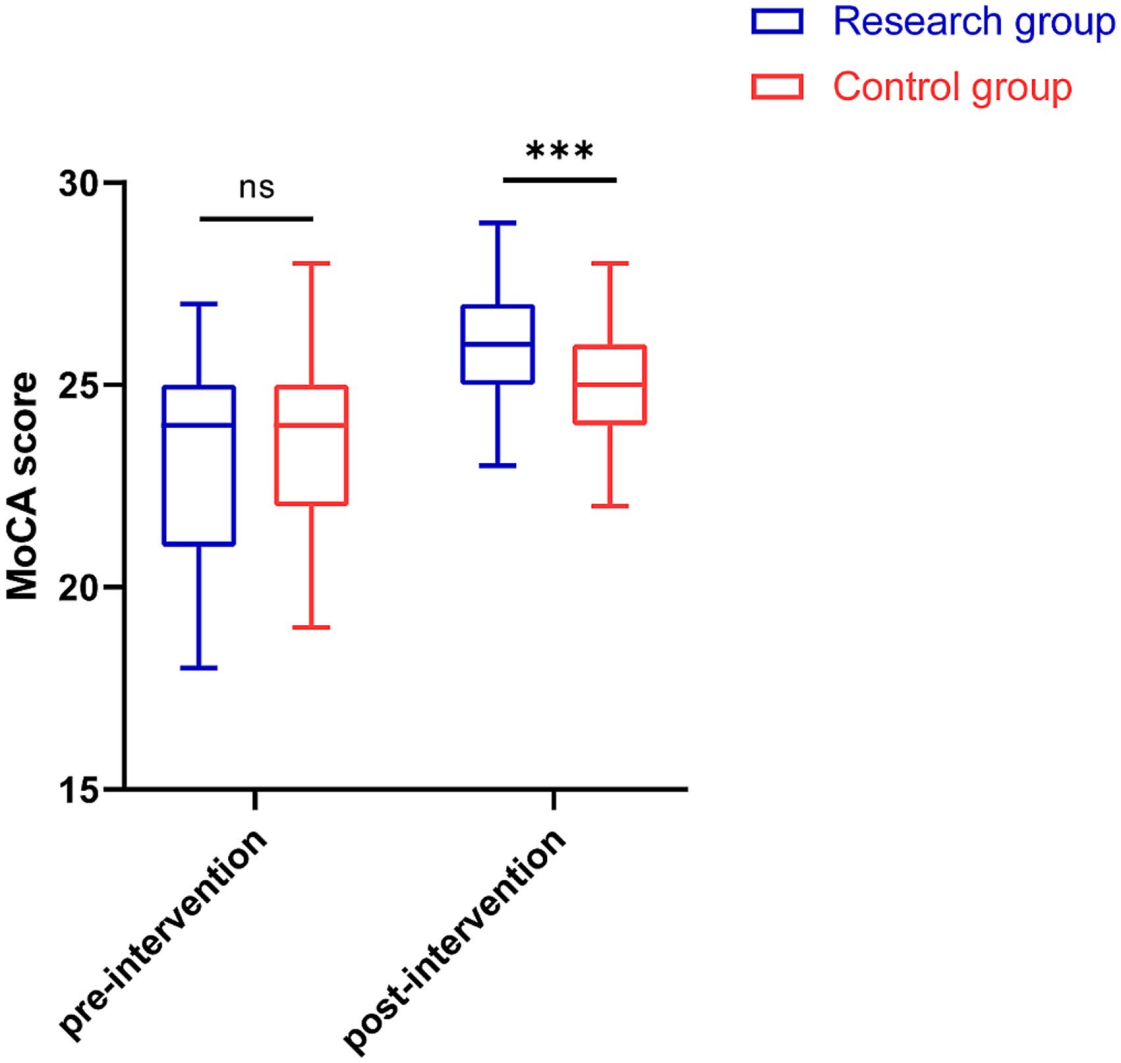
MoCA scores before and after the intervention in both groups. MoCA, Montreal cognitive assessment. ***p < 0.001.

Before intervention, the MoCA scores of the research group and the control group were 23.16 ± 2.57 and 23.40 ± 2.07, respectively, and the difference between the two groups was not statistically significant (*p* = 0.645). After the intervention, MoCA scores increased in both groups, with the research group having significantly higher MoCA scores than the control group (26.23 ± 1.45 vs. 25.05 ± 1.17, *p* < 0.001; Figure 1).

### Psychological resilience

**Figure 2:**
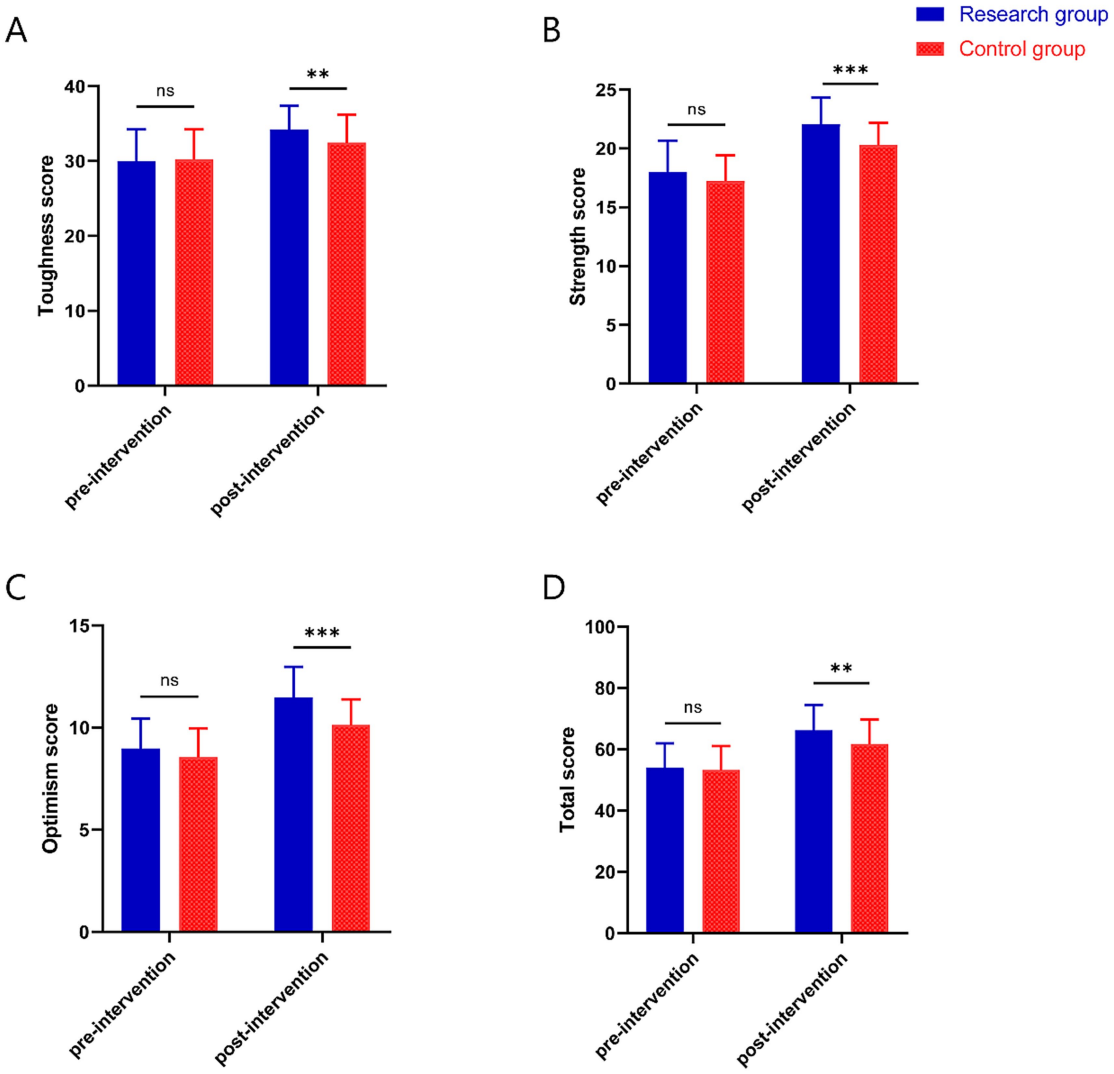
Psychological resilience scores before and after the intervention in both groups. **(A)** toughness score, **(B)** strength score, **(C)** optimism score, **(D)** total score. **p < 0.01 and ***p < 0.001.

Before intervention, there were no significant differences between the two groups in toughness (29.98 ± 4.26 vs. 30.23 ± 4.00, *p* = 0.775), strength (17.98 ± 2.70 vs. 17.23 ± 2.17, *p* = 0.163), optimism (8.98 ± 1.46 vs. 8.56 ± 1.42, *p* = 0.181), and total score (54.07 ± 7.92 vs. 53.28 ± 7.85, *p* = 0.643). Toughness, strength, optimism, and total score increased in both groups after the intervention, and toughness (34.26 ± 3.13 vs. 32.47 ± 3.75, *p* = 0.018), strength (22.07 ± 2.29 vs. 20.28 ± 1.92, *p* < 0.001), optimism (11.49 ± 1.49 vs. 10.14 ± 1.25, *p* < 0.001), and total score (66.26 ± 8.27 vs. 61.79 ± 7.93, *p* = 0.012) were all significantly higher in the research group than in the control group (Figure 2).

### Self-efficacy

Before intervention, the GSES scores of the research group and the control group were 22.40 ± 2.32 and 22.09 ± 2.07, respectively, and the difference between the two groups was not statistically significant (*p* = 0.525). After the intervention, self-efficacy scores increased in both groups, with the GSES scores of the research group significantly higher than those of the control group (30.19 ± 3.27 vs. 26.37 ± 2.31, *p* < 0.001; Table 2).

**Table 2:** Comparison of psychological resilience between the two groups (mean ± SD).

Classification	Before intervention	After intervention
Research group(*n* = 43)	22.40 ± 2.32	30.19 ± 3.27
Control group(*n* = 43)	22.09 ± 2.07	26.37 ± 2.31
*T*	0.638	6.250
*p*-value	0.525	<0.001

### Cancer-caused fatigue

Before intervention, there was no statistically significant difference in the data of the indicators of PFS between the two groups (*p* > 0.05). After the intervention, behavioral fatigue (4.02 ± 1.28 vs. 4.61 ± 1.37, *p* = 0.045), emotional fatigue (3.28 ± 1.39 vs. 3.93 ± 1.40, *p* = 0.033), physical fatigue (3.81 ± 1.30 vs. 4.47 ± 1.37, *p* = 0.026), and cognitive fatigue (4.07 ± 1.39 vs. 4.72 ± 1.37, *p* = 0.031) scores were reduced and were significantly lower in the research group than in the control group (Table 3).

**Table 3:** Comparison of cancer-caused fatigue between the two groups (mean ± SD).

Classification		Research group (*n* = 43)	Control group (*n* = 43)	*t*	*p*-value
Behavioral	Before intervention	4.98 ± 1.32	4.84 ± 1.21	0.511	0.611
	After intervention	4.02 ± 1.28	4.61 ± 1.37	2.036	0.045
Emotionally	Before intervention	4.54 ± 1.18	4.44 ± 1.03	0.390	0.698
	After intervention	3.28 ± 1.39	3.93 ± 1.40	2.165	0.033
Somatic	Before intervention	4.84 ± 1.25	4.72 ± 1.32	0.420	0.676
	After intervention	3.81 ± 1.30	4.47 ± 1.37	2.265	0.026
Cognitive	Before intervention	5.16 ± 1.29	5.12 ± 1.18	0.175	0.862
	After intervention	4.07 ± 1.39	4.72 ± 1.37	2.192	0.031

### Sleep quality

Comparison of the data of PSQI indicators between the two groups of patients before intervention, the difference was not statistically significant (*p* > 0.05). After the intervention, sleep quality (1.51 ± 0.67 vs. 1.98 ± 0.74, *p* = 0.003), sleep speed (1.58 ± 0.70 vs. 2.05 ± 0.75, *p* = 0.004), sleep duration (1.30 ± 0.60 vs. 1.63 ± 0.76, *p* = 0.030), sleep efficiency (1.14 ± 0.56 vs. 1.56 ± 0.77, *p* = 0.005), sleep disorders (1.58 ± 0.67 vs. 2.05 ± 0.75, *p* = 0.003), hypnotic drug (0.47 ± 0.51 vs. 0.79 ± 0.77, *p* = 0.023), day functioning (0.74 ± 0.49 vs. 1.14 ± 0.92, *p* = 0.015), and total score (8.63 ± 1.59 vs. 11.12 ± 1.31, *p* < 0.001) in the research group were significantly lower than those of the control group (Table 4).

**Table 4:** Comparison of sleep quality between the two groups (mean ± SD).

Classification		Research group (*n* = 43)	Control group (*n* = 43)	*t*	*p*-value
Sleep quality	Before intervention	2.42 ± 0.55	2.37 ± 0.54	0.399	0.691
	After intervention	1.51 ± 0.67	1.98 ± 0.74	3.060	0.003
Sleep speed	Before intervention	2.40 ± 0.54	2.33 ± 0.57	0.585	0.560
	After intervention	1.58 ± 0.70	2.05 ± 0.75	2.967	0.004
Sleep duration	Before intervention	1.95 ± 0.79	1.93 ± 0.80	0.136	0.892
	After intervention	1.30 ± 0.60	1.63 ± 0.76	2.212	0.030
Sleep efficiency	Before intervention	1.79 ± 0.78	1.84 ± 0.84	0.267	0.790
	After intervention	1.14 ± 0.56	1.56 ± 0.77	2.895	0.005
Sleep disorder	Before intervention	2.33 ± 0.61	2.21 ± 0.64	0.866	0.389
	After intervention	1.58 ± 0.67	2.05 ± 0.75	3.037	0.003
Hypnotic drug	Before intervention	1.14 ± 0.60	1.09 ± 0.97	0.267	0.790
	After intervention	0.47 ± 0.51	0.79 ± 0.77	2.312	0.023
Day function	Before intervention	1.65 ± 0.95	1.70 ± 1.04	0.217	0.829
	After intervention	0.74 ± 0.49	1.14 ± 0.92	2.495	0.015
Total score	Before intervention	14.19 ± 1.14	14.09 ± 1.13	0.380	0.705
	After intervention	8.63 ± 1.59	11.12 ± 1.31	7.916	<0.001

## Discussion

This study investigated the intervention effect of MBSR on POCD and psychological resilience in lung cancer patients through a randomized controlled trial. The results showed that the research group receiving MBSR was significantly better than the control group receiving only conventional care in terms of cognitive function, psychological resilience, self-efficacy, cancer-caused fatigue, and sleep quality. Postoperative cognitive dysfunction is a common complication in lung cancer patients, and the mechanism may be related to surgical trauma, systemic inflammatory response, and neuroinflammatory activation. Relevant studies have found that mindfulness training interventions can enhance executive control by strengthening functional connectivity between the prefrontal cortex and the fronto-parietal regions responsible for coordinating executive functions (28, 29). The results of this study showed that the MoCA score of the research group was significantly higher than that of the control group after the intervention (*p* < 0.001), indicating that MBSR has a positive intervention effect on POCD. This finding aligns with the systematic review by Gotink et al., indicating that an 8-week MBSR training program can induce enhanced prefrontal functional connectivity, thereby improving attention and executive function (30). We speculate that exercises in MBSR, such as mindful breathing, body scanning, and sitting meditation, may improve cognitive function through multiple pathways, including enhancing patients’ ability to allocate attentional resources and reducing external interference; regulating the autonomic nervous system and reducing the level of stress hormones, thus reducing neuroinflammatory damage to the hippocampus and prefrontal lobe; and reducing the occupancy of cognitive resources by anxiety and depression through emotion regulation. In addition, the significant increase in self-efficacy in the research group may have further promoted the patients’ active participation in cognitive rehabilitation training, forming a positive cycle. However, these mechanisms necessitate further validation through future research. Psychological resilience is a core psychological resource for cancer patients to cope with the challenges of the disease. In this study, the total CD-RISC scores of the research group were significantly higher than those of the control group after the intervention (*p* = 0.012), with the improvements in the ‘resilience’ and ‘optimism’ dimensions being particularly prominent. This result supports Finkelstein-Fox’s finding that positive thinking training can help patients reconstruct adversity cognition by enhancing emotional acceptance (31). Specifically, the self-exploration module in weeks 7–8 of the MBSR enhanced patients’ resilience to the illness by guiding them to confront their anxieties and fears and reduce emotional avoidance behaviors. In addition, the family’s joint participation in the positive thinking sessions may have strengthened the effects of the intervention through social support. Increased psychological resilience not only directly relieves patients’ psychological stress but may also indirectly promote postoperative recovery by reducing the suppressive effects of chronic stress on immune function through hypothalamic–pituitary–adrenal (HPA) axis regulation (32). Cancer-induced fatigue and sleep disturbances are common physical and psychological symptoms in lung cancer patients after surgery, and they often exacerbate each other. The results of the study showed that the PFS scores and the total PSQI scores of the research group were significantly better than those of the control group (*p* < 0.05). This may be related to the following mechanisms: positive thinking exercises activate the parasympathetic nervous system and reduce the metabolic hyperactivity triggered by sympathetic arousal, thus reducing the feeling of physical fatigue; the enhancement of the ability to regulate emotions reduces the interference of ‘ruminative thinking’ on sleep; and walking meditation and other dynamic exercises promote endorphin secretion through moderate exercise and improve the daytime functioning of the body. Notably, improved sleep quality may further alleviate cognitive fatigue, creating a virtuous circle. This finding echoes Bower’s study, suggesting that MBSR has multi-targeted intervention benefits in cancer symptom management (33).

In this study, we extended MBSR to patients after lung cancer surgery and systematically evaluated its effect on POCD and psychological resilience for the first time. The family participatory MBSR intervention design enhanced the promoting effect of social support on psychological resilience. Based on the results of the study, MBSR is recommended to be included in the standardized nursing procedures for postoperative rehabilitation of lung cancer patients. Specific measures include introducing the concept of mindfulness in preoperative education to help patients establish positive coping strategies; forming multidisciplinary teams to conduct group-based mindfulness courses; developing family participation models and strengthening social support through training of family members; and using digital tools such as mindfulness apps to provide ongoing interventions for discharged patients. In addition, there is a need to strengthen mindfulness skills training for healthcare workers to ensure normative and compliant implementation of interventions.

### Limitations

Despite the positive results of this study, several limitations were also encountered. First, the small sample size and single-center design may limit the generalizability of the results. Second, this study selected a relatively healthy subgroup of postoperative lung cancer patients, which may limit the generalizability of the findings to patients with advanced disease or poorer prognosis. Third, blinding of outcome assessors was not implemented in this study, which may introduce detection bias in subjective, patient-reported outcomes. Fourth, the intervention period was 8 weeks, and there is a lack of long-term follow-up data to assess the persistence of the effect. Finally, the results relied on a scale-based assessment without objective measures such as neuroimaging or biomarkers. Future studies may further optimize the design by conducting multicenter large-sample randomized controlled trials; extending the follow-up period to 6–12 months to observe the long-term effects; combining fMRI or EEG techniques to reveal the neural mechanisms by which MBSR affects cognitive function; and exploring personalized intervention options such as mobile-based positive thinking apps to improve accessibility.

## Conclusion

MBSR can effectively improve cognitive function, enhance psychological resilience, and alleviate cancer-related fatigue and sleep disorders in post-operative lung cancer patients. Its mechanism of action involves multiple pathways such as neuroplasticity regulation, stress response inhibition, and social support reinforcement. Despite some limitations, this study provides high-quality evidence for the use of MBSR in lung cancer rehabilitation and highlights the direction for future research. It is recommended that clinical practitioners integrate positive thinking interventions into the multimodal rehabilitation programs, taking into account the individual patient needs to comprehensively improve quality of life and prognosis.

## Data Availability

The raw data supporting the conclusions of this article will be made available by the authors, without undue reservation.
